# Comparison of the efficacy, safety and postoperative quality of life between modified side overlap anastomosis and double-tract anastomosis after laparoscopic proximal gastrectomy

**DOI:** 10.1007/s13304-024-01830-6

**Published:** 2024-04-03

**Authors:** Chu-Ying Wu, Wen-Jin Zhong, Kai Ye

**Affiliations:** Department of Gastrointestinal Surgery, The Second Affiliated Hospital of Fujian Medical University, No. 950 Donghai Street, Fengze District, Quanzhou, 362000 Fujian Province China

**Keywords:** Proximal gastrectomy, Digestive tract reconstruction, Double-tract anastomosis, Side overlap anastomosis, Quality of life

## Abstract

**Purpose:**

To compare the surgical safety and postoperative quality of life (QOL) between side overlap anastomosis (SOA) and double-tract anastomosis (DTA) after laparoscopic proximal gastrectomy (LPG).

**Methods:**

This retrospective cohort study included 43 patients with proximal gastric cancer (PGC) who underwent LPG and were admitted to the Second Affiliated Hospital of Fujian Medical University between August 2020 and December 2022 were in. Their clinical and follow-up data were collected. The patients were divided into the modified SOA (mSOA) (*n* = 20) and DTA (*n* = 23) groups based on the anastomosis methods used. The main outcome measures included the QOL of patients 1 year after surgery, and the evaluation criteria were based on the postgastrectomy syndrome assessment scale. Secondary outcome measures included intraoperative and postoperative conditions, postoperative long-term complications and nutritional status 3, 6 and 12 months after surgery.

**Results:**

No significant differences were observed in intraoperative and postoperative conditions (*P* > 0.05) between the mSOA and DTA groups. The mSOA group showed a decreased incidence of reflux esophagitis 1 year after surgery compared with the DTA group (*P* < 0.05), and no statistically significant differences were noticed between the two groups in terms of other postoperative complications (*P* > 0.05). The mSOA group showed better QOL when compared with the DTA group (*P* < 0.05). No significant differences were recorded in postoperative nutritional status between the two groups (*P* > 0.05).

**Conclusion:**

The efficacy and safety of LPG with mSOA for PGC were comparable. When compared with the DTA group, the mSOA group seems to show reduced incidence of gastroesophageal reflux and improved QOL, which makes mSOA one of the ideal surgical methods for PGC.

## Introduction

The incidence of proximal gastric cancer (PGC) has increased over the past decade despite the decreased overall incidence of gastric cancer [1]. With the development of the concept of function-preserving surgery, PG is only advised for the treatment of early gastric cancer of the upper stomach, with at least 1/2 of the stomach preserved [2, 3]. An increasing number of digestive tract reconstructions, including double-tract anastomosis (DTA), tube gastroesophageal anastomosis, side-overlap anastomosis (SOA) and double-muscle flap anastomosis (Kamikawa), are now being used after PG in clinical practice. However, the anti-reflux mechanism and advantages and disadvantages of these procedures remain controversial, and no optimal digestive tract reconstruction methods have been established after PG [4, 5]. SOA, which was developed by Yamashita et al. in 2017 [6], is relatively easy to perform and effectively prevents postoperative gastroesophageal reflux. It continued to be modified by Yamashita et al. [6] to make the anastomotic site more accurate and stable and improve its reflux prevention mechanism. When compared with conventional esophagogastrostomy, DTA can prevent the direct reflux of gastric acid into the esophagus and maintain a good nutritional state after surgery [7–9].

The current comparative studies on digestive tract reconstructions following PG mostly focused on conventional esophagogastrostomy, DTA and double-muscle flap anastomosis [10, 11]. However, a comparison between mSOA and DTA has not been reported. On this basis, the present study, which involved retrospective analysis, was designed to explore and compare the efficacy and safety and the effect of mSOA and DTA after laparoscopic proximal gastrectomy (LPG) on postoperative QOL to provide further references for the selection of digestive tract reconstruction methods for the treatment of PGC.

## Materials and methods

### Study subjects

This work is a retrospective cohort study. The inclusion criteria comprised patients (1) diagnosed with primary gastric adenocarcinoma via preoperative endoscopic pathological biopsy, with stage I or II gastric cancer according to the 8th edition of the American Joint Committee on Cancer, (2) with a tumor located in the upper 1/3 of the stomach, as confirmed by preoperative computed tomography and other imaging examinations; (3) treated with PG, DTA or tube gastroesophageal anastomosis as the digestive tract reconstruction method. The exclusion criteria included patients who (1) received preoperative neoadjuvant therapy, (2) were complicated with tumors of other sites, (3) failed to receive R0 excision, and (4) with incomplete postoperative follow-up data.

A total of 43 patients with proximal gastric adenocarcinoma admitted to the Gastrointestinal Surgery Center of the Second Affiliated Hospital of Fujian Medical University between August 2020 and December 2022 were retrospectively selected in accordance with the above eligibility criteria, and their clinicopathological data were collected. The patients were divided into the mSOA (*n* = 20) and DTA (*n* = 23) groups based on the anastomosis methods used. Baseline data were compared between the two groups, but no statistical significance was observed (Table 1). All patients and their families provided informed consent before the surgical procedure. This study was reviewed and approved by the Ethics Committee of the Second Affiliated Hospital of Fujian Medical University.

**Table 1:** Baseline data

	Modified side-overlap anastomosis group	Double-tract anastomosis group	*p* value
Number	20	23	
Age (years)	65.5 (60.8, 71.8)	70 (66, 76)	0.095
Sex			1.000
Male	17	19	
Female	3	4	
BMI (kg/m^2^)	21.9 ± 3.3	22.1 ± 2.8	0.885
American Society of Anesthesiologists grade			0.106
I	10	6	
II	10	17	
Tumor T stage			0.142
1	14	11	
2	6	12	
Tumor N stage			0.434
0	16	16	
1	4	7	
Tumor TNM stage			0.281
I	16	15	
II	4	8	
Maximum tumor diameter (cm)	2.4 ± 1.0	2.4 ± 1.1	0.923

### Surgical procedures

**Fig. 1:**
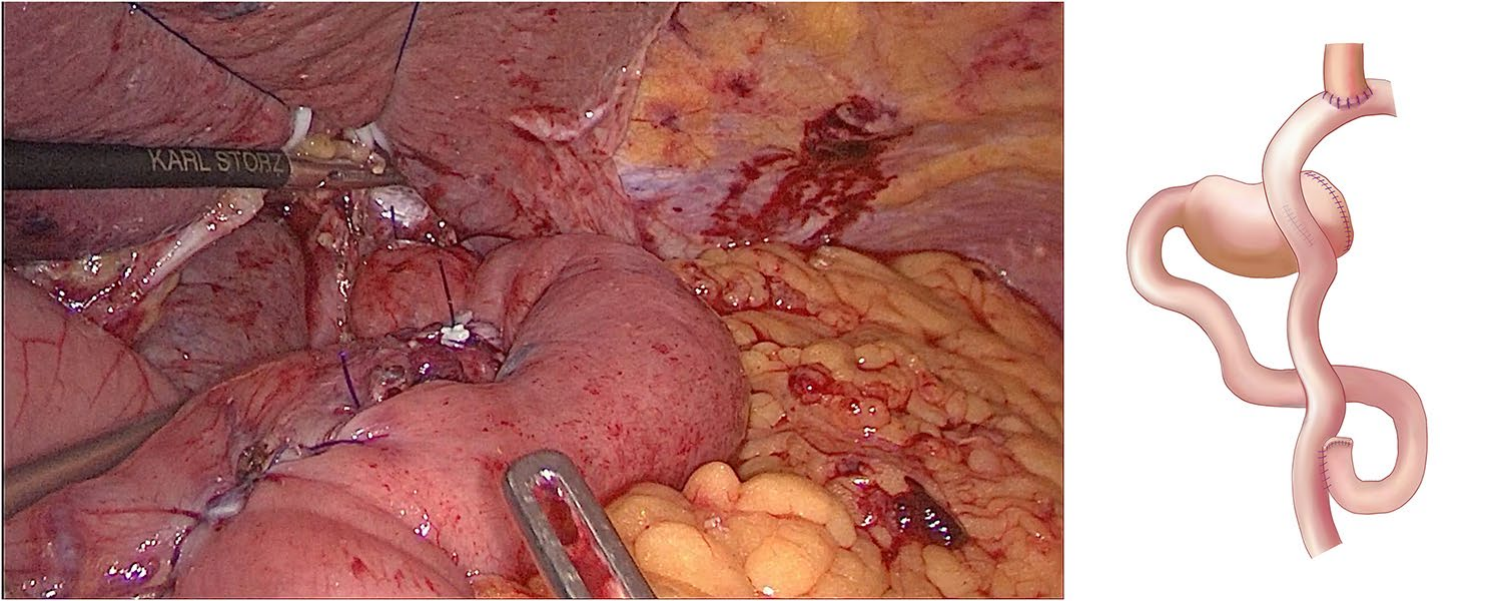
Double-tract anastomosis

All procedures were performed laparoscopically. All patients in the two groups underwent D1 and D1 + D2 lymphadenectomy in accordance with the requirements of PG [3]. Perigastric vessels and the omentum were dissected, and the esophagus was dissected at the proximal end as required. The methods for distal digestive tract reconstruction for the two groups of patients were as follows:

(1). DTA group: the stomach was dissected at a distal site > 5 cm from the edge of the tumor, and the jejunum was dissected 15–20 cm away from the Treitz ligament. As shown in Fig. 1, esophagojejunal, gastrojejunal and jejunojejunal anastomoses were conducted between the distal jejunal stump and esophagus using a linear stapler between the distal remnant stomach and jejunum 10–15 cm below the esophagojejunal anastomosis and on the proximal jejunal stump 25–30 cm below the gastrojejunal anastomosis, respectively.

(2). mSOA group: The lower segment of the esophagus was exposed to a length of ≥ 5 cm, and the esophagus was transected near the cardia using an endoscopic linear stapler. Intraoperative gastroscopy or rapid frozen pathological examination can be performed to ensure safe surgical margins. The abdominal segment of the esophagus should be reserved as much as possible, and a thread should be placed in the middle of the lower edge of the esophageal stump to facilitate subsequent traction. Bilateral diaphragmatic cruses were opened to expand the operating space. Subsequent suture and fixation of the esophageal stump were guided by marking the dorsal side 5 cm from the esophageal stump with gentian violet.

A small median incision (5 cm) was made under the xiphoid process, and the stomach was pulled out to determine the tumor location. The stomach was dissected at the proximal end with a linear stapler 3 cm away from the distal end of the tumor, and the stump was fortified by intermittent suture. The cutting line should be as perpendicular to the long axis of the stomach as possible to preserve the remnant stomach volume. A 5 cm longitudinal mark was made with gentian violet at the median of the anterior wall of the remnant stomach to be anastomosed with the esophagus. The remnant stomach was then returned to the abdominal cavity to reestablish the pneumoperitoneum.

**Fig. 2:**
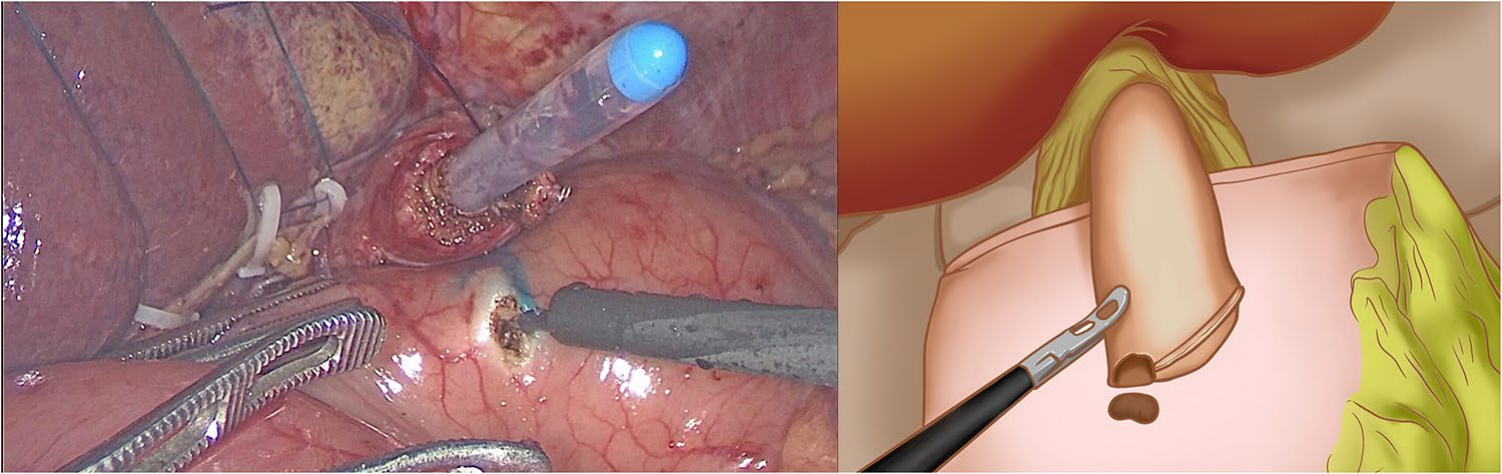
A small incision was made on the right wall of the lower esophageal stump using an electric hook and another at the lower end of the gentian violet mark on the anterior wall of the remnant stomach

**Fig. 3:**
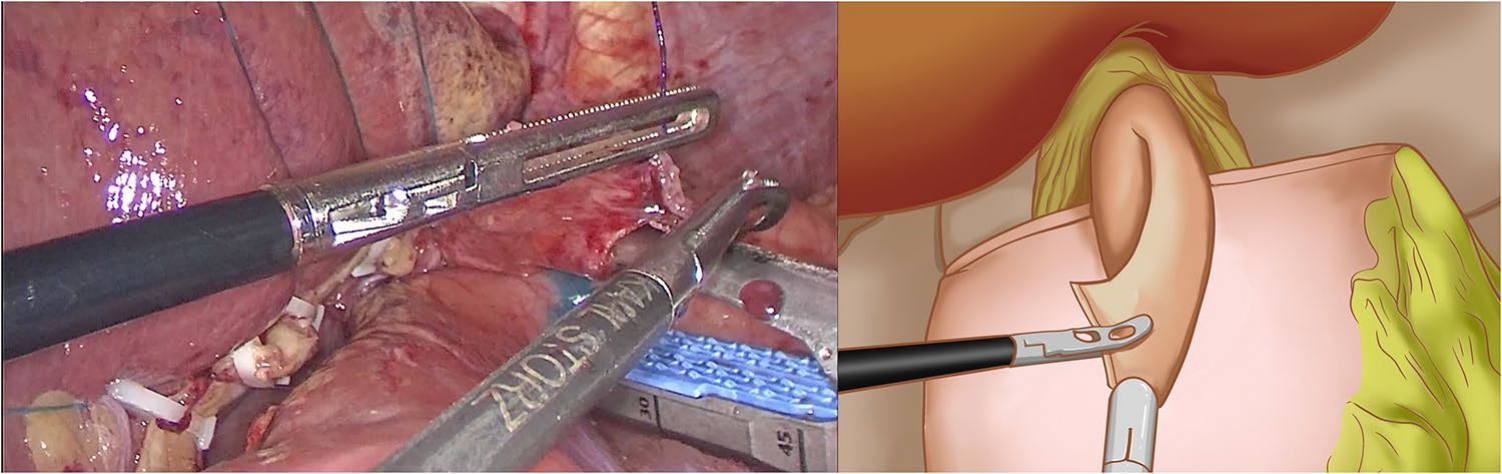
A 45 mm linear stapler was inserted through the opening of the esophagus and remnant stomach to conduct a side-to-side anastomosis between the esophagus and remnant stomach

**Fig. 4:**
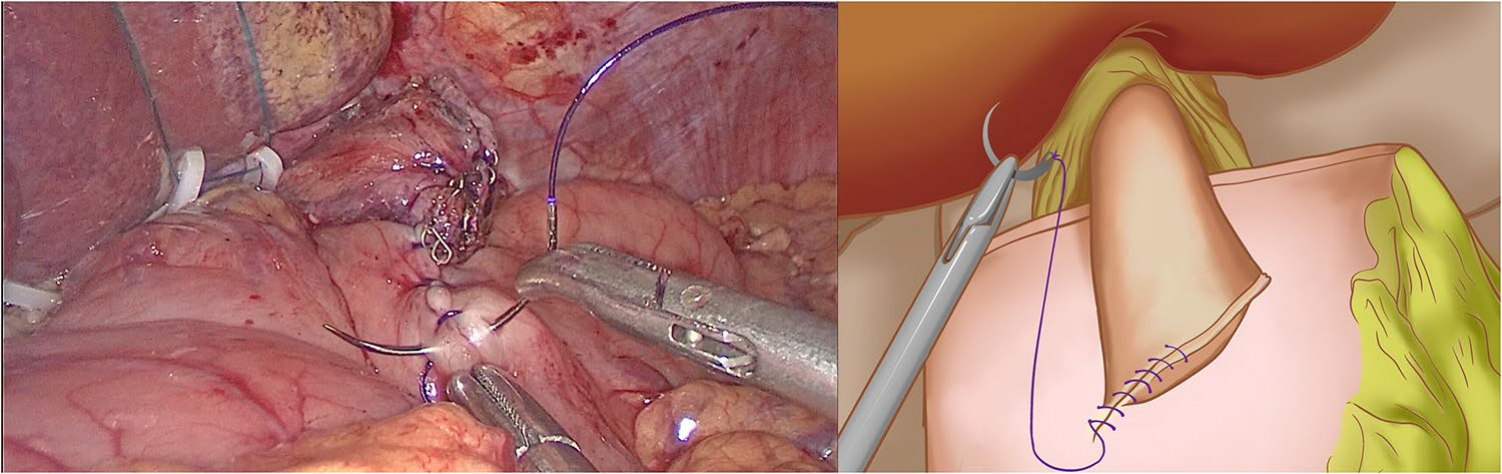
The entry hole was closed with a longitudinal continuous barbed suture

**Fig. 5:**
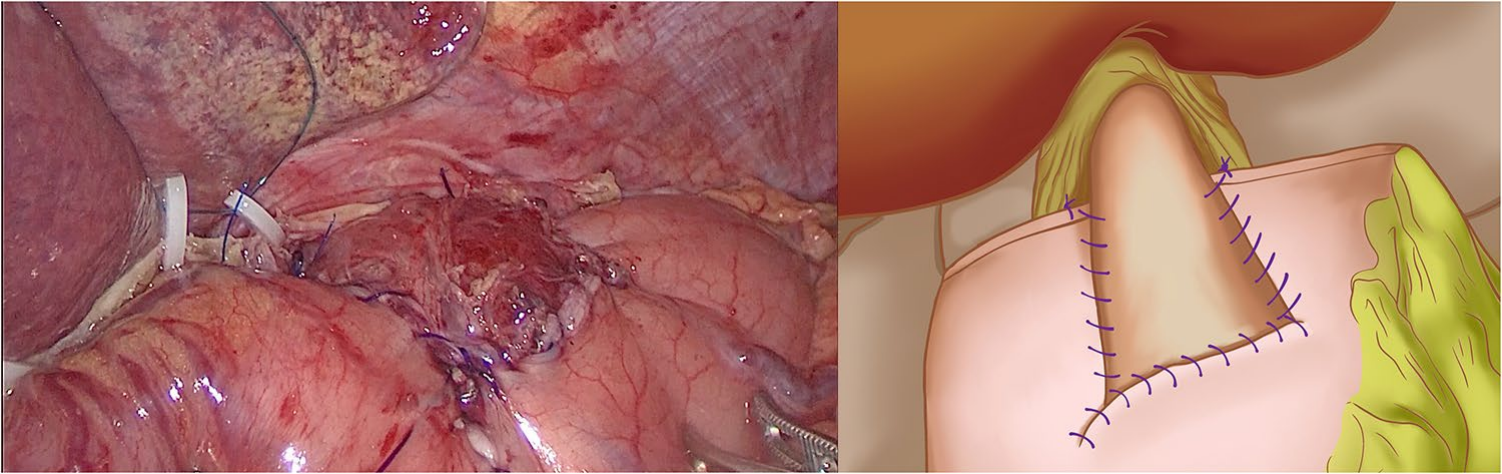
Modified side-overlap anastomosis

At the gentian violet mark, the center of the upper edge of the remnant stomach was fixed by a continuous suture of the dorsal side of the lower esophagus. A barbed suture was used to ensure that the lower esophagus accurately overlapped and was fixed at the center of the anterior wall of the remnant stomach during subsequent esophagogastric anastomosis. Under the guidance of a gastric tube, a small incision was made on the right wall of the lower esophageal stump using an electric hook and another at the lower end of the gentian violet mark on the anterior wall of the remnant stomach (Fig. 2). The left side of the esophageal stump was lifted and rotated counterclockwise by 90°. With the gentian violet mark on the anterior wall of the remnant stomach as a guide, the long axis of the esophagus was paralleled and overlapped with the long axis of the stomach. A 45 mm linear stapler was inserted through the opening of the esophagus and remnant stomach to conduct a side-to-side anastomosis between the esophagus and remnant stomach (Fig. 3). The entry hole was closed with a longitudinal continuous barbed suture (Fig. 4). Blue staplers were used, and a thicker staple cartridge was used on the thick parts of the stomach or esophageal wall. The lower margin of the esophageal stump was continuously fixed by suture, with the anterior wall of the remnant stomach from the left side of the lower esophagus, using a barbed suture to embed the cutting edge of the lower esophagus. The left and right ends of the remnant stomach were suture-fixed to the left and right diaphragmatic cruses to reconstruct an artificial gastric fundus. The left and right sides of the lower esophagus were continuously suture-fixed with the anterior wall of the remnant stomach for 270° embedding to ensure a tight fit between the lower esophagus and the anterior wall of the remnant stomach. The reconstruction of the digestive tract was completed (Fig. 5).

**Fig. 6:**
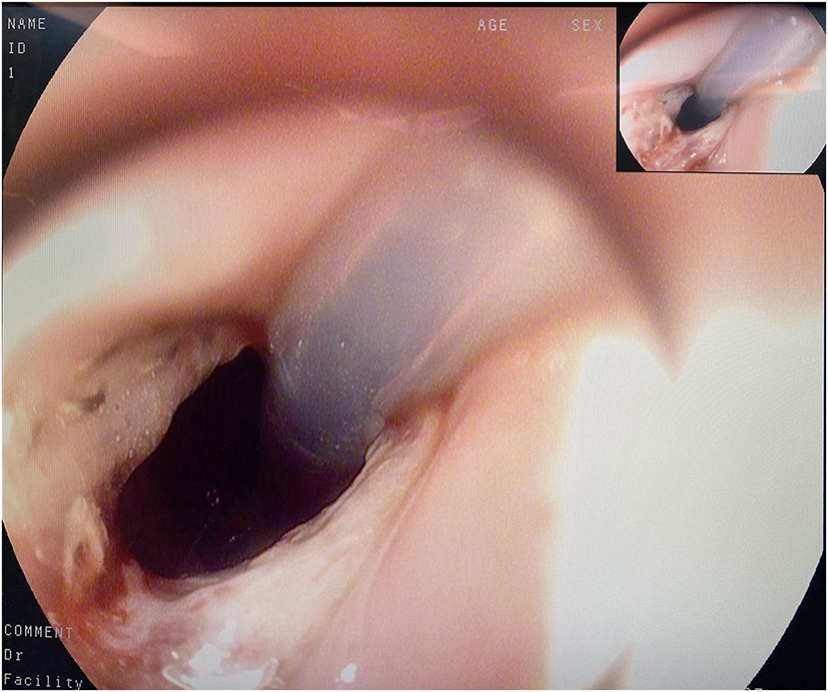
A narrow and elongated anastomosis was observed through intraoperative gastroscopy

**Fig. 7:**
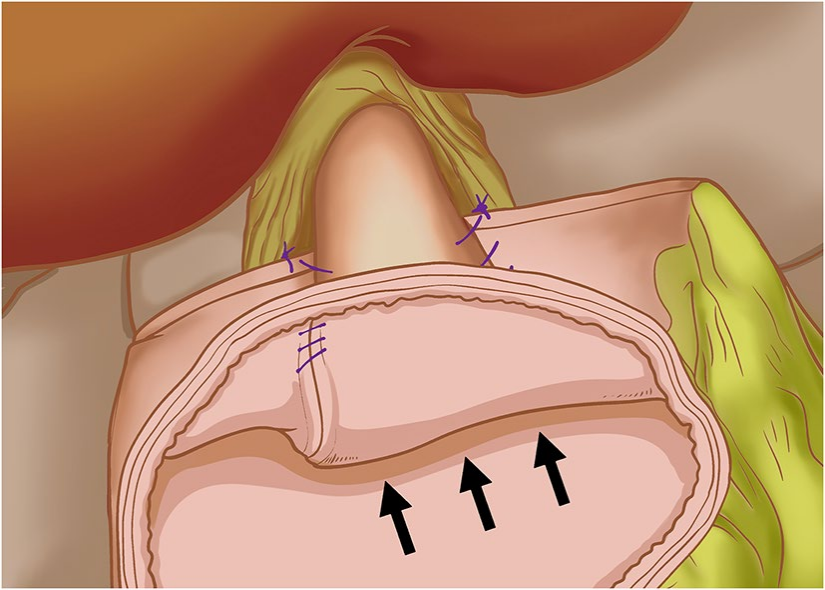
When the pressure of the artificial gastric fundus increased, the posterior wall of the esophagus on the anastomosis adhered to the anterior wall to prevent gastroesophageal reflux

Finally, the anastomotic site was carefully examined for bleeding, stricture or incomplete closure through intraoperative gastroscopy, and a narrow and elongated anastomosis was observed (Fig. 6). The reconstructed anastomosis formed a valve-like structure. When the pressure of the artificial gastric fundus increased, the posterior wall of the esophagus on the anastomosis adhered to the anterior wall to prevent gastroesophageal reflux (Fig. 7).

### Outcome measures and evaluation criteria

(1) Outcome measures: The primary outcome measures included the QOL of patients 1 year after surgery, and the secondary ones comprised intraoperative and postoperative conditions, postoperative long-term complications and nutritional status 6 and 12 months after surgery.

(2) Evaluation criteria: (a) QOL: The postgastrectomy syndrome assessment scale (PGSAS-45) designed by the Japanese Postgastrectomy Syndrome Working Party (JPGSWP) was used to determine the intensity of various symptoms after gastrectomy and their impact on patients’ daily lives. The scale mainly consists of symptoms, living status and QOL domains. Related problems in different domains were graded based on their severity. High scores on the subscales of body mass change, food intake per meal and meal quality and the total scores of physical health and mental health indicated a good condition; for the other items, high scores suggested a poor condition [12]. (b) Postoperative long-term complications: Gastroesophageal reflux, anastomotic stricture, intestinal obstruction and delayed gastric emptying were observed 1 year after surgery. Gastroesophageal reflux was evaluated via gastroscopy, and the severity of reflux esophagitis was graded using the Los Angeles scale [13]. (c) Nutritional status: body mass index (BMI), Nutritional Risk Screening 2002 (NRS2002) [14] and Patient-Generated Subjective Global Assessment (PG-SGA) scores [15] were used to evaluate the nutritional status of the patients.

### Follow-up method

The patients were followed up for 1 year, and physical symptoms, living status, QOL recovery and nutritional status were collected 6 and 12 months after surgery through outpatient visits, telephone calls or questionnaires. The deadline for follow-up was December 2023.

### Statistical analysis

All data were analyzed using SPSS 26.0. Measurement data with normal distribution are presented as *x* ± *s*, and two independent sample t tests were used for comparison between groups. Measurement data with non-normal distribution are presented as M(Q1,Q3), and the Mann–Whitney *U* test was used for comparison between groups. Enumeration data are presented as *n* (%), and the *χ*^2^ test was used for comparison of nonhierarchical data between groups. The Mann–Whitney *U* test was used for the comparison of hierarchical data between groups. Differences with a *p*-value of < 0.05 were considered statistically significant.

## Results

### Comparison of intraoperative and postoperative conditions between the two groups

**Fig. 8:**
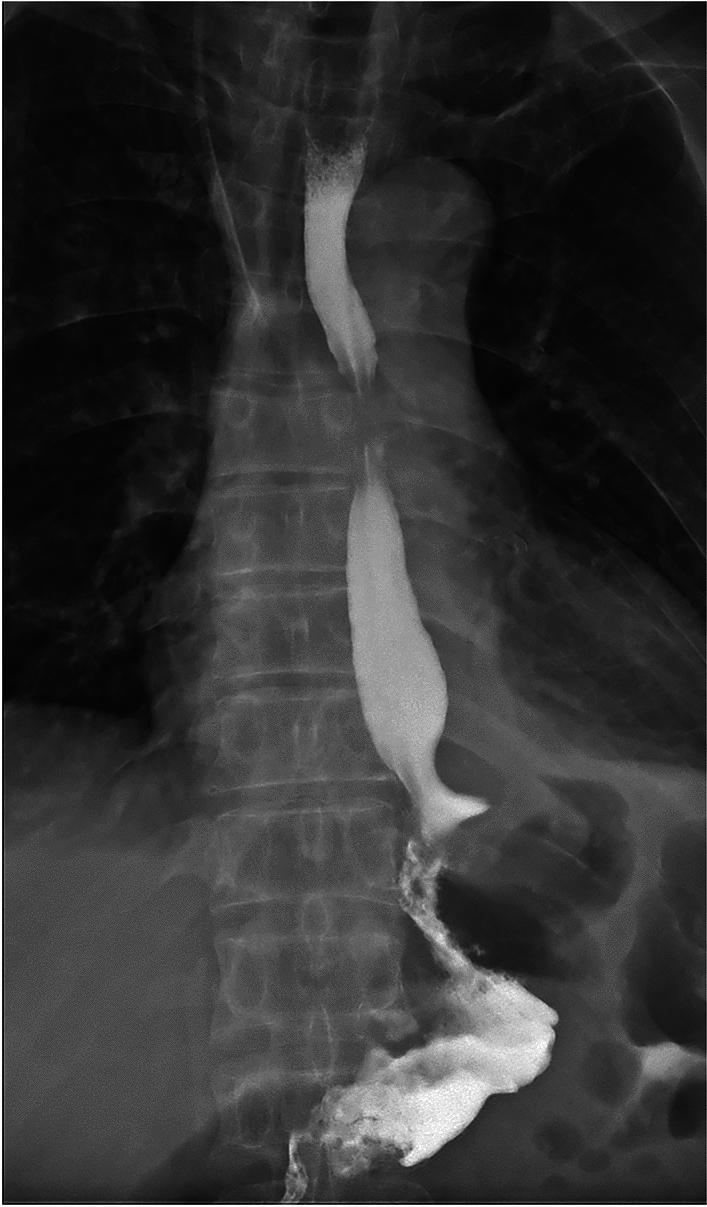
Radiography of the upper digestive tract were shown in the mSOA group

**Fig. 9:**
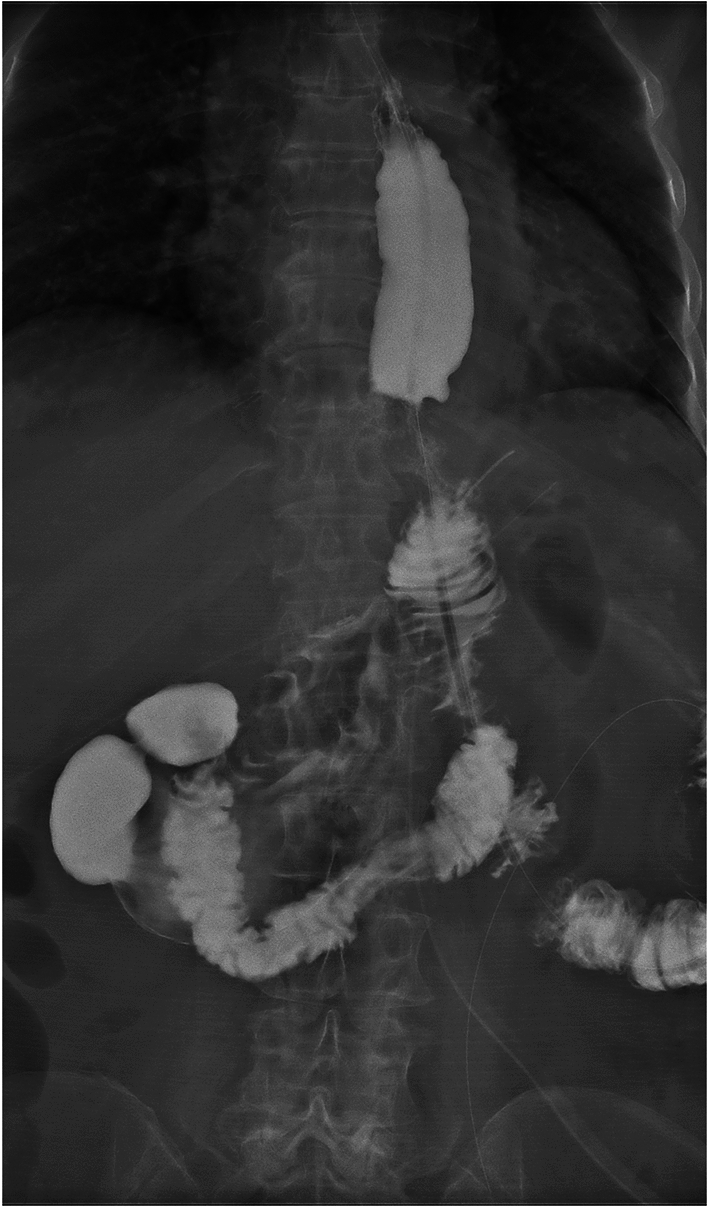
Double tract images were observed in 19 patients of the DTA group

**Fig. 10:**
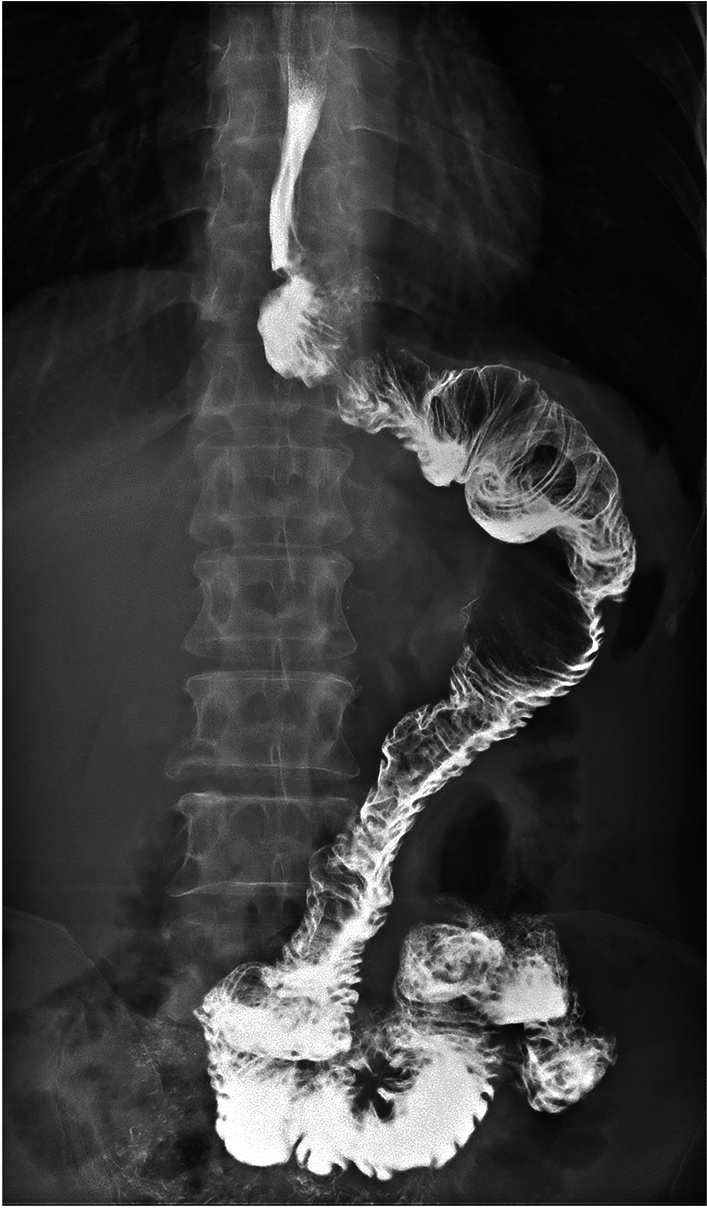
Single tract images were observed in 4 patients of the DTA group

All patients in the two groups underwent R0 resection and successfully completed the surgery. No significant differences were observed between the two groups in terms of operation time, intraoperative blood loss, the number of lymph node dissections, digestive tract reconstruction time, hospital stay and incidence of short-term postoperative complications (*p* > 0.05 for all comparisons; Table 2). All patients with anastomotic bleeding received endoscopic hemostasis treatment, and other complications were resolved after symptomatic conservative treatment. No patient underwent a second surgery. All patients completed radiography of the upper digestive tract 7 days after surgery, and the results for the mSOA group are shown in Fig. 8. In the DTA group, double and single tract images were observed in 19 (82.6%; Fig. 9) and 4 (17.4%; Fig. 10) patients, respectively.

**Table 2:** Intraoperative and postoperative conditions

	Modified side-overlap anastomosis group	double-tract anastomosis group	*p* value
Number	20	23	
Operation time (min)	197.5 (190.0, 207.0)	187 (177, 198)	0.103
Digestive tract reconstruction time (min)	80.5 (77.3, 89.8)	83 (79, 96)	0.214
Intraoperative blood loss (ml)	21.9 ± 3.0	21.2 ± 4.1	0.518
The number of lymph node dissections	24.8 ± 11.0	20.4 ± 10.3	0.190
Hospital stay	8.0 (7.0, 8.8)	8 (8, 9)	0.069
Short-term postoperative complications	3	2	0.868
Anastomotic hemorrhage	1	1	
Anastomotic fistula	0	0	
Gastroplegia	1	0	
Pneumonia	0	1	
Incision infection	1	0

### Comparison of postoperative QOL between the two groups

Compared with the DTA group, the mSOA group exhibited better gastroesophageal reflux, eating discomfort and total symptom score in the physical symptom domain, postoperative symptoms, meals and daily lives in the living status and QOL domains and anal exhaust 1 year after surgery, and the differences were statistically significant (*p* < 0.05 for all comparisons; Table 3).

**Table 3:** Postoperative Quality of life

	Modified side-overlap anastomosis group	Double-tract anastomosis group	*p* value
Number	20	23	
Symptoms			
Esophageal reflux subscale	3.0 ± 1.2	4.1 ± 1.3	0.006
Abdominal pain subscale	1.7 (1.3, 3.0)	2.0 (1.3, 3.3)	0.468
Meal-related distress subscale	2.8 ± 1.0	3.7 ± 1.1	0.003
Indigestion subscale	2.3 (2.3, 3.0)	2.5 (2.3, 3.5)	0.458
Diarrhea subscale	1.3 (1.3, 2.0)	1.3 (1.7, 2.0)	0.764
Constipation subscale	1.3 (1.3, 1.7)	1.3 (1.3, 1.7)	0.737
Dumping subscale	1.3 (1.3, 1.3)	1.3 (1.3, 1.3)	0.890
Total symptom score	2.9 ± 1.1	3.7 ± 1.4	0.045
Other outcome measures (symptom)			
Increased flatus	2.5 (2, 3)	4 (3, 5)	0.036
Loose stools	1 (1, 2)	1 (1, 2)	0.976
Living status			
Change in body weight (%)^a^	12.1 ± 4.6	12.9 ± 4.3	0.556
Ingested amount of food per meal^a^	4 (4, 5)	4 (4, 5)	0.966
Necessity for additional meals	3.5 (3, 4)	3.5 (3, 4)	0.926
Quality of ingestion subscale^a^	4.0 (3.3, 4.7)	3.3 (3.0, 3.7)	0.026
Ability for working^a^	1 (1, 2)	1 (1, 2)	0.988
Quality of life			
Dissatisfaction with symptoms	1 (1, 2)	2 (2, 3)	0.002
Dissatisfaction at the meal	1 (1, 2)	2 (2, 3)	< 0.001
Dissatisfaction at working	1.0(1.0,1.8)	1 (1, 2)	0.330
Dissatisfaction for daily life subscale	1.7 (1.3, 2.0)	2.3 (2.0, 3.0)	0.005
Physical component summary^a^	81.1 ± 5.4	80.9 ± 4.9	0.909
Mental component summary^a^	90.8 ± 6.0	90.2 ± 4.6	0.725

In items or subscales with ^a^, higher score indicates better condition. In items or subscales without ^a^, higher score indicates worse condition

### Comparison of long-term postoperative complications between the two groups

The mSOA group showed decreased incidence and severity of reflux esophagitis compared with the DTA group 1 year after surgery, with statistically significant differences (*p* < 0.05 for all comparisons). No statistically significant differences were noted in the incidences of postoperative anastomotic stricture, intestinal obstruction and delayed gastric emptying between the two groups (*p* > 0.05 for all comparisons; Table 4). Anastomotic stricture was reported in 1 patient of each group, and it improved after endoscopic anastomotic dilation treatment. The prognoses of other patients in the two groups who experienced long-term complications were favorable after conservative treatment.

**Table 4:** Long-term postoperative complications

	Modified side-overlap anastomosis group	Double-tract anastomosis group	*p* value
Number	20	23	
Reflux esophagitis	2	9	0.029
Grade A	2	5	
Grade B级	0	4	
Anastomotic stricture	1	1	1.000
Intestinal obstruction	0	1	1.000
Delayed gastric emptying	1	2	1.000

Comparison of the postoperative nutritional status between the studied groups

All patients were followed up 3, 6 and 12 months after surgery, and no statistically significant differences were found in the BMI, NRS2002 score and PG-SGA score of the mSOA and DTA groups (*p* > 0.05; Table 5).

**Table 5:** Comparison of the postoperative nutritional status between the studied groups

	Modified side-overlap anastomosis group	Double-tract anastomosis group	*p* value
Number	20	23	
BMI at 3 months after surgery (kg/m^2^)	21.3±2.6	21.5±2.6	0.0.868
BMI at 6 months after surgery (kg/m^2^)	21.5±2.9	21.4±2.6	0.947
BMI at 12 months after surgery (kg/m^2^)	22.0±2.5	22.3±2.6	0.751
NRS 2002 score at 3 months after surgery	2(1.25, 2)	2(1,2)	0.330
NRS 2002 score at 6 months after surgery	2(2,2)	2(1,2)	0.575
NRS 2002 score at 12 months after surgery	1.5(1,2)	1.5(1,2)	0.888
PG-SGA score at 3 months after surgery	2(1.25,2)	2(1,2)	0.491
PG-SGA score at 6 months after surgery	2(1,2)	2(1,2)	0.575
PG-SGA score at 12 months after surgery	2(1,2)	2(1,2)	0.855

## Discussion

Digestive tract reconstruction after radical resection of PGC has been a concern among clinicians. In the past, TG with Roux-en-Y esophagojejunostomy was the first choice of treatment for clinicians; however, patients easily suffered from malnutrition after this procedure [16]. Although conventional PG with gastroesophageal anastomosis can preserve the distal stomach and improve nutritional status to a certain extent, the remnant stomach fails to control the cardia, which leads to postoperative reflux and impaired QOL [17]. The anti-reflux mechanism is an important parameter in the evaluation of digestive tract reconstruction after PG. Studies have reported the established anti-reflux effect of SOA and DTA and increased QOL and nutritional status compared with conventional gastroesophageal anastomosis [6, 18]. However, determining which is better between SOA and DTA in terms of anti-reflux mechanism, postoperative nutritional status and QOL remains controversial.

The conventional SOA, which requires a linear stapler alone, is relatively easy to perform with and inexpensive. The key point is that during side-to-side anastomosis of the esophagus and remnant stomach, the linear stapler is rotated counterclockwise and fired to anastomose the side wall of the esophagus and anterior wall of the remnant stomach. The opposite side wall of the esophagus is fixed closely to the anterior wall of the remnant stomach. Then, the remnant stomach is suture-fixed to the left and right diaphragmatic cruses to reconstruct an artificial gastric fundus. When the pressure of the artificial gastric fundus increases, the end of the esophagus closes to prevent reflux. However, Yamashita et al. reported an anastomotic shift to the lesser curvature of the remnant stomach in some of the 27 patients receiving SOA, which resulted in the insufficient overlap between the lower esophagus and anterior wall of the remnant stomach and consequent ineffective closure of the esophagus when the pressure of the artificial gastric fundus increased. This finding can be explained by the mismatch between the long axis of the esophagus, the long axis of the remnant stomach and the rotation axis of the linear stapler at the time of firing.

On this basis, Yamashita et al. [6] modified the side overlap method to enhance the stable anti-reflux mechanism. In the present study, the modified side overlap method was applied in LPG, and the preliminary experience related to this modified method included the following: (1) Compared with the conventional side overlap method, the most significant improvement of the mSOA was the replacement of side-to-side anastomosis between the left side wall of the lower esophagus and anterior wall of the remnant stomach with side-to-side anastomosis between the right side wall of the lower esophagus and anterior wall of the remnant stomach. Implementation of the conventional side overlap method mainly depends on the subjective judgement and experience of surgeons and is associated with anastomotic shift and suboptimal therapeutic effects. In mSOA, the esophagus was rotated counterclockwise by 90° to parallelize the long axis of the esophagus to the middle long axis of the remnant stomach. A mark made in advance was used to guide the subsequent side-to-side anastomosis of the esophagus and remnant stomach to ensure accurate fitting and effectively prevent the shifting of the long axes of the esophagus, the remnant stomach and the linear stapler during anastomosis. (2) The left and right diaphragmatic cruses were opened to expand the operation space and establish an artificial gastric fundus by lifting the remnant stomach and increasing the length of the exposed esophagus, which effectively solved the problems of retraction after esophageal resection and anastomosis difficulties after excessive esophageal resection and created favorable conditions for subsequent anastomosis and the construction of anti-reflux mechanism. (3) Similar to fundoplication, the left and right sides of the lower esophagus were suture fixed with the anterior wall of the adjacent remnant stomach for 270° embedding, which increased the pressure of the gastric fundus and improved the anti-reflux mechanism. (4) The lower esophageal incisal margin was embedded to prevent postoperative anastomotic leakage. The reconstructed anastomosis formed a valve-like structure. When the pressure of the artificial gastric fundus was increased, the posterior wall of the esophagus on the anastomosis adhered to its anterior wall to prevent gastroesophageal reflux. Compared with the 25 mm circular stapler, a long and wide anastomosis formed in the mSOA. Shifts in the long axis of the esophagus, the remnant stomach and the linear stapler should be avoided during anastomosis to prevent postoperative anastomosis stricture. If a shift occurs, the anastomosis can be widened intraoperatively by changing the suture direction of the common opening.

In addition to SOA, PG with DTA is a common technique. This procedure was first reported by Aikou et al.[19] in 1988. After proximal gastric dissection, Roux-en-Y esophagojejunostomy was performed, and a side-to-side anastomosis was conducted between the remnant stomach and jejunum to establish an anti-reflux barrier to reduce the incidence of postoperative gastroesophageal reflux. Ji et al. observed that the incidence of reflux esophagitis in patients undergoing PG with DTA reached 8% 1 year after operation [20]; Nomura et al. reported reflux esophagitis in 6.7% of patients during endoscopy [21].

Innovations of the present study include a systematic evaluation of QOL after PG with mSOA versus DTA. Compared with the previous studies, the PGSAS-45 scale designed by the JPGSWP was used in the present study to evaluate the postoperative QOL of patients in the two groups. This scale is the only comprehensive questionnaire suitable for patient assessment after different gastrectomy and reconstruction operations [12]. The results of the present study show that in the physical symptom domain, gastroesophageal reflux symptoms and eating discomfort 1 year after surgery were improved in the mSOA group compared with those in the DTA group. In the postoperative QOL domain, the mSOA group showed increased satisfaction with daily living compared with that in the DTA group. During the follow-up period, gastroesophageal reflux and eating discomfort considerably affected the life of patients, and mSOA effectively reduced the development of these symptoms. Patients in the DTA group were more likely to experience increased anal exhaust compared with those in the mSOA group, which was possibly due to the rapid entry of food into the jejunum. These results indicate that the overall postoperative QOL of patients was increased in the mSOA group compared with that in the DTA group.

The postoperative nutritional status is also an important factor in the selection of digestive tract reconstruction methods after PG. In PG with mSOA, the gastroduodenal pathway is preserved, and the physiological and anatomical structures of the stomach are maintained. In addition, pepsinogen and intrinsic factors secreted by the remnant stomach promote the digestion and absorption of food [22]. DTA allows entry of food to the distal digestive tract through two pathways. The preserved remnant stomach can promote the transport and mixing of bile and food, and a portion of food can directly enter the jejunum, which can alleviate delayed gastric emptying or stasis caused by vagotomy. Theoretically, both reconstruction methods can improve the nutritional status of patients after surgery [18]. Food is directly taken into the jejunum without entering the remnant stomach in some patients undergoing DTA, which leads to insufficient nutrition absorption [9]. The results of BMI and nutritional status assessment in the present study showed no significant difference in the postoperative nutritional status between patients undergoing mSOA and DTA after PG, and both digestive tract reconstruction methods can ensure good postoperative nutritional status.

Tumor treatment is based on the safety and feasibility of surgery and radical resection of tumors. All patients in the present study underwent R0 resection with negative surgical margins, as evidenced by postoperative pathology. No significant difference was observed in the number of lymph node dissections between the two groups. Compared with TG, the number of lymph nodes for examination was reduced [23]. Given the less distal lymph node metastasis of PGC, PG is still suitable for stage I and II gastric cancer [24]. To improve the accuracy of lymph node staging, one must completely dissect the lymph nodes in accordance with surgical requirements and increase the number of lymph nodes for examination. The results of the present study reveal that the surgical conditions and incidence of perioperative complications were similar between the two groups. Moreover, the incidence of anastomotic leakage in the DTA group did not increase, although the number of anastomoses increased during this procedure.

This study has several limitations: (1) The inclusion of only 43 patients represents a relatively small sample size, diminishing the statistical power to some extent. (2) Given the retrospective nature of the study, there exists a possibility of selection bias, despite the absence of significant baseline differences. The study exclusively enrolled early proximal gastric cancer cases with ASA classes 1–2, while excluding those undergoing neoadjuvant therapy. Further research is needed to determine whether the findings are applicable to patients with higher ASA classes or locally advanced proximal gastric cancer receiving neoadjuvant therapy or other types. (3) The analysis did not incorporate patient occupation and education levels, introducing a potential bias in reporting postoperative QOL results. (4) The relatively short follow-up period, limited to 1 year postoperatively, precluded a comparison of long-term complications such as anastomotic stricture rates, esophageal reflux rates, and patient survival. The durability of the short-term advantages observed in the SOA group for long-term benefits remains uncertain, necessitating additional follow-up in subsequent phases. Future endeavors will focus on augmenting sample size, extending the follow-up duration, and initiating prospective single-center studies to further validate the study's findings, paving the way for subsequent multi-center investigations.

In summary, mSOA seems to be superior to DTA in terms of QOL for the treatment of stage I and II PGC. It can substantially improve postoperative gastroesophageal reflux symptoms and eating discomfort, and its efficacy and safety are comparable to those of DTA. The results of the present study can provide references for surgeons during the selection of surgical methods.

## Data Availability

All data generated or analyzed during this study are included in this published article.
